# Improving care experiences for premenstrual symptoms and disorders in the United Kingdom (UK): a mixed-methods approach

**DOI:** 10.1186/s12913-024-12140-3

**Published:** 2025-01-14

**Authors:** E. L. Funnell, N. A. Martin-Key, S. Bahn

**Affiliations:** Cambridge Centre for Neuropsychiatric Research, Department of Chemical Engineering and Biotechnology, University of Cambridge, Cambridge, UK

**Keywords:** Premenstrual symptoms, Premenstrual disorders, Premenstrual dysphoric disorder, Healthcare

## Abstract

**Background:**

Poor care experiences are reported for premenstrual disorders, which may result in negative outcomes such as distress, reduced healthcare engagement, and delays to diagnosis. This research aimed to explore healthcare experiences for premenstrual symptoms in the United Kingdom and identify areas for potential improvements based on participant responses.

**Method:**

An online survey was delivered, with participants recruited via social media. Experiences of care were explored using quantitative and qualitative questions. Quantitative data were explored using descriptive statistics, with group differences investigated using Mann–Whitney U tests and chi-square tests as appropriate. Qualitative data regarding care improvements was explored using inductive thematic analysis.

**Results:**

The sample included 339 participants who completed at least 97% of the survey, reported premenstrual symptoms in consecutive cycles, and had sought formal help for these symptoms. Mean age was 34.66 (SD = 5.88), with the majority identifying as women (*n* = 332, 97.94%) and white/Caucasian (*n* = 311, 91.74%). 75.22% (*n* = 255) felt that care was poor. 44.25% (*n* = 150) felt their symptoms were not taken seriously. 37.76% perceived a lack of healthcare professional knowledge (*n* = 128). The majority did not receive recommendations of non-formal sources of help (i.e., websites, support groups; 84.96%, *n* = 288) or lifestyle changes (74.63%, *n* = 253). Better care experiences were associated with healthcare professionals taking symptoms seriously (U = 1383.00, *p* < .001), higher perceived healthcare professional knowledge (U = 1370.50, *p* < .001), and receiving recommendations of non-formal sources of help (X^2^ = 48.251, df = 1, *p* < .001, Φc = .382) or lifestyle changes (X^2^ = 7.849, df = 1, *p* = .005, Φc = .152). Thematic analysis revealed 8 aspects of care improvement: Empathetic care provision; Healthcare professional education, understanding, and research; Comprehensive symptom assessment and investigations; Diagnosis; Professional support and treatment provision; Signposting or referral to additional resources or sources of help; Wider healthcare system improvements; and Patient role/voice and preferences.

**Conclusions:**

Poor care experiences for premenstrual symptoms in the UK are characterised by dismissive attitudes and perceived lack of knowledge. Improved training provision for healthcare professionals is required to address this and other aspects of care identified by qualitative analysis. However, research is needed to identify appropriate methods to deliver training. Utilisation of standardised screening tools and patient-centred communication will likely ensure comprehensive assessments and reduce self-advocacy burdens.

**Supplementary Information:**

The online version contains supplementary material available at 10.1186/s12913-024-12140-3.

## Introduction

Premenstrual symptoms are exceedingly common in women and other people assigned female at birth [1]. These symptoms encompass physical symptoms (e.g., headaches, bloating), psychological symptoms (e.g., tearfulness, irritability), and behavioural symptoms (e.g., social withdrawal, emotional reactivity) occurring in the luteal phase of the menstrual cycle. These symptoms, whilst cyclical, can adversely impact daily life even without a formal clinical diagnosis [2]. Premenstrual disorders such as premenstrual syndrome (PMS) and premenstrual dysphoric disorder (PMDD) are characterised by more severe symptoms and an accompanying substantial impact on functioning and quality of life, which is generally not observed for premenstrual symptoms. Comprehensive symptom tracking and evaluation by a healthcare provider are often essential for determining if premenstrual symptoms meet the threshold for a diagnosis of premenstrual disorder, with diagnostic criteria set out for PMDD in the diagnostic and statistical manual of mental health disorders. Such disorders are frequent, with PMS estimated to impact 50% of the menstruating population [3] and estimates of PMDD ranging from 3 to 8% [4], although these estimates may be inflated due to provisional diagnoses, with the pooled point prevalence in community samples found to be 1.6% [5]. Premenstrual disorders have wide-ranging impacts, including but not limited to, decreased productivity [6] and increased suicidality risk [7, 8]. However, it is worth noting that individuals who lack a formal PMDD diagnosis may still experience significant or distressing premenstrual symptoms that lead them to seek professional care.

First-line treatment for premenstrual symptoms and disorders are lifestyle changes such as increasing exercise, improving sleep quality, and reducing alcohol and smoking [9]. Further treatments include psychological (e.g., cognitive behavioural therapy) and pharmacological (e.g., antidepressants, combined oral contraceptives) therapies [10] accessed through contact with healthcare services [11]. Unfortunately, dismissive healthcare professional (HCP) attitudes are extremely commonplace in women’s health [12]. This may result in individuals having to engage in long-term self-advocacy to receive care [12] and delays to receiving treatment. It may also limit the ability of help-seeking individuals to participate in shared-decision making with a clinician regarding treatment [12]. For premenstrual disorders, individuals report feeling as though they had to prove the severity of their symptoms to a clinician in order to be taken seriously [13]. Unfortunately, this dismissiveness of premenstrual symptoms can result in delays to diagnosis of PMDD [13], with additional risks of misdiagnosis [13, 14]. Delays to diagnosis have wide-ranging impacts on the individual [15] and can result in the development of secondary mental health symptoms [16].

Although previous work has investigated experiences of healthcare provision for PMDD in the United Kingdom (UK) [16] and the United States (US) [13], to our knowledge, no recent research has investigated care experiences more broadly for premenstrual symptoms. Since individuals without a formal PMDD diagnosis can still experience significant or distressing premenstrual symptoms that may prompt them to seek professional help, it is important to broadly examine experiences of formal care for premenstrual symptoms, regardless of the severity of or diagnosis associated with those symptoms. Therefore, we sought to examine healthcare experiences of individuals in the UK who have specifically sought formal help for premenstrual symptoms. Additionally, given the potential negative consequences associated with poor care experiences for premenstrual symptoms and disorders, it is important to determine how care can be improved. To this end, we also aimed to explore how healthcare experiences could be improved from the perspective of previous help-seekers.

## Methods

### Participants

Participants were recruited using free posts on Twitter and paid advertisements on Facebook and Instagram between January 2024 and February 2024. Recruitment materials invited potential participants to complete an online survey, and specified the focus of the study was experiences of help-seeking and care for premenstrual symptoms. Free posts on Twitter did not have a clearly defined target population; however, as they were shared from the CCNR account, they likely reached individuals within our research network. Hashtags related to the study, such as “premenstrual”, “PMS”, “PMDD”, and “research” were used to increase visibility and maximise the likelihood that the recruitment materials were seen by individuals engaging with these topics. Paid advertisements on Facebook and Instagram were targeted to individuals in the UK who identified as female and are aged 18–45. Gender identity was used as a proxy for assigned sex at birth due to limitations in advertisement targeting data. This age group was chosen primarily due to its relevance to menopause, though the advertisements were also delivered to individuals outside of this range. The estimated size of reach through these recruitment channels was 110,000 individuals.

Inclusion criteria were: (1) 18 years or older, (2) having a strong comprehension of the English language, (3) be assigned female at birth, (4) currently experiencing premenstrual symptoms, (5) not currently pregnant, in the perimenopause, or post-menopausal, and (6) not diagnosed with any gynaecological conditions (e.g., endometriosis, polycystic ovary syndrome). Participants were required to confirm that they met the inclusion criteria when consenting to participate in the study, and before commencing the survey. Participation in the study was on a voluntary basis.

## Materials and procedures

A mixed-methods, anonymous online survey was developed for the current study and delivered using the survey software Qualtrics XM®. The survey took 10–20 min to complete, and was adaptive in nature so only relevant questions were displayed based on previous answers. The survey was designed in consultation with an experienced consultant psychiatrist (SB). The survey was broadly comprised of three questions domains: 1) sociodemographic information and premenstrual symptoms, 2) healthcare, diagnosis, and treatment perspectives, and 3) barriers and facilitators to accessing formal care. For the purposes of the current study, only data from domains 1 and 2 were included (to see the questions from domains 1 and 2 which are relevant to the current study, see Supplementary Document A1 (Supplementary information)).

All participants were asked about sociodemographic characteristics. Participants were asked about their formal care experiences including the type of HCPs they saw and the overall perceived quality of care they had received. Participants rated their perception of the overall quality of care they received on a four-point scale, with the scale points: 1 = “Very poor”, 2 = “Poor”, 3 = “Good”, 4 = “Very good”. Perceived seriousness of premenstrual symptoms by HCP(s) was rated on a four-point scale, with the scale points: 1 = “Not at all seriously”, 2 = “Slightly seriously”, 3 = “Moderately seriously”, and 4 = “Very seriously”. Participants were also asked to rate how knowledgeable they perceived the HCP to be about premenstrual symptoms and disorders, using a four-point scale including the points: 1 = “Not at all”, 2 = “Slightly”, 3 = “Moderately”, and 4 = “Very”. For this question there was also an answer option “I am not sure”. Participants were additionally asked if they had received any recommendations of lifestyle changes or other non-formal sources of help (e.g., leaflets, websites, peer support groups).

Participants were also asked the optional open text question: “In your opinion, what could have improved your care experience?”.

Additionally, participants were asked if they have received a formal diagnosis of PMDD. If yes, these participants were asked about their experience of receiving this diagnosis.

### Ethical approval

The study was approved by the University of Cambridge Psychology Research Ethics Committee (approval number PRE.2023.117). All participants provided informed consent to participate before starting the survey.

### Data analysis

Descriptive data analyses (i.e., means and standard deviations, frequencies and percentages) were analysed and processed in Excel, version 2206 (Microsoft Office 365). Figures were created using Excel version 2206 and PowerPoint version 2206 (Microsoft Office 365).

Group differences in care interaction characteristics between those who rated their formal care as “Good” or “Very good” and those who rated their formal care as “Poor” or “Very poor” were explored. Group differences in ordinal data were explored using Mann–Whitney U tests in order to determine whether there was a difference in the perceived seriousness and knowledge of the healthcare professional between those who rated their care as “Good” or “Very good” and those who rated their care as “Poor” or “Very poor”. Group differences in categorical variables were explored using chi-square tests, and effect sizes were calculated as Cramer’s V (φc; small ≤ 0.10, medium ≤ 0.30, large ≤ 0.50; [17]). For all group differences analyses, “I am not sure” responses for questions with this answer option were excluded. Tests for group differences were conducted in SPSS (version 29.0.1.1).

Open text data was analysed using thematic analysis with an inductive, data-driven approach using the Braun and Clarke framework [18]. Thematic analysis was conducted by a research assistant (EF) and a senior research associate (NMK). Both authors have previous experience of thematic data analysis. One author read and re-read the open text data to establish data familiarisation before writing a codebook (EF). This codebook was reviewed by a second author (NMK) to ensure all relevant codes were included and any necessary amendments were made. Open text data was coded against the code book under blinded conditions by two authors (EF and NMK). Following this, the data was unblinded and coding allocation was compared, with any discrepancies being discussed until agreement was reached. After coding was agreed, the codes were grouped into themes by two authors (EF and NMK) under blinded conditions. Again, following unblinding, themes were compared with any discrepancies being discussed until a final theme list was agreed. Any free text data which included “No” (or synonyms), “Not applicable” (or synonyms), or responses that were considered ambiguous or non-specific by both authors (e.g., “More options”) were labelled as not applicable and excluded from thematic analysis. Intercoder reliability was reported against the evaluation framework developed by Cofie et al. [19] (Supplementary Table A1 (Supplementary information)). Please note, no changes have been made to the illustrative quotes; any spelling or grammatical errors appear as provided by the participants. Quantitative and qualitative data were analysed separately, but qualitative results were used to contextualize and expand upon the quantitative results.

## Results

Participants who had completed at least 97% of the survey, endorsed premenstrual symptoms in consecutive menstrual cycles, and had sought help from a HCP specifically for premenstrual symptoms (*N* = 339) were included in the analyses.

### Sociodemographic data

The mean age of the sample was 34.66 (SD = 5.88; range = 18–50). The majority of the sample identified as women (*n* = 332, 97.94%) and were white/Caucasian (*n* = 311, 91.74%). The majority held at least an undergraduate degree (*n* = 276, 81.42%) and were in paid employment (*n* = 284, 83.78%). See Supplementary Table A2 (Supplementary information) for full summary of the sociodemographic characteristics.

### Healthcare experiences

The majority of those who had sought formal help had done so from a primary care provider (e.g., general practitioner, primary care physician, family physician, family doctor; 95.87%, *n* = 325), with 65.49% (*n* = 222) having only seen one type of HCP.

**Fig. 1:**
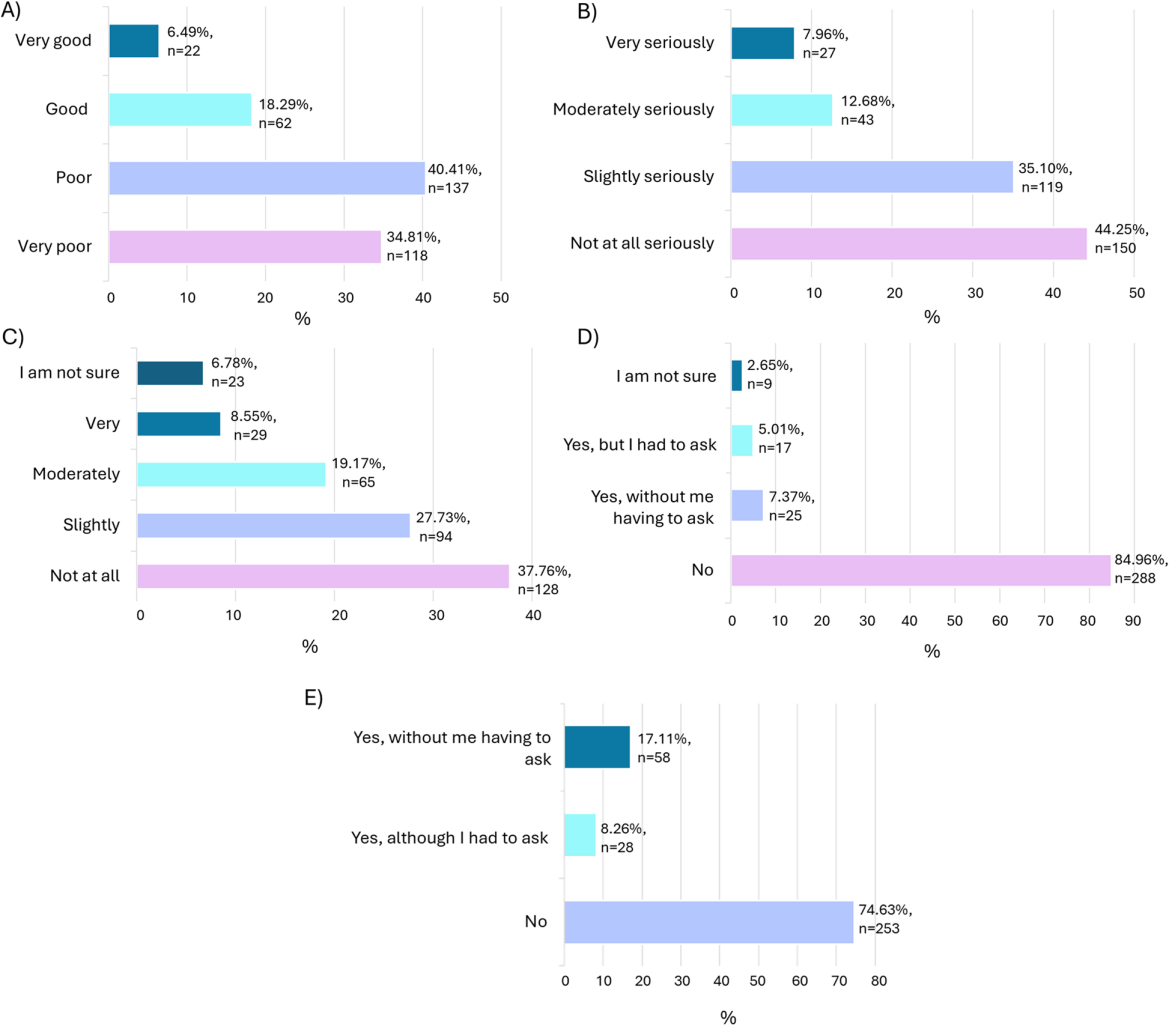
Perceptions of formal health care. Key. **A**) Overall perceived care quality, **B**) Perceived seriousness of symptom consideration, **C**) Perceived HCP knowledge about premenstrual symptoms and disorders, **D**) Whether alternative recommendations or referrals of non-formal sources of help were provided by HCP, **E**) Whether recommendations of lifestyle changes were provided by HCP

Of the sample, 75.22% (*n* = 255; Fig. 1a) felt that the overall quality of their care was poor. 44.25% (*n* = 150; Fig. 1b) felt their symptoms were not taken seriously, and many perceived a lack of HCP knowledge regarding premenstrual disorders (37.76%, *n* = 128; Fig. 1c). The majority did not receive referrals to or recommendations for additional non-formal sources of help (i.e., websites, support groups; 84.96%, *n* = 288; Fig. 1d). The majority did not receive any recommendation of lifestyle changes which could be made to help manage premenstrual symptoms by a HCP (74.63%, *n* = 253; Fig. 1e).

When comparing perceived care quality, the group that rated their overall care quality as "Good" or “Very good” felt that healthcare providers took their symptoms more seriously (mean = 3.06, SD = 0.77; U = 1383.00, *p* < 0.001) compared to the group who rated their overall care as “Poor” or “Very poor” (mean = 1.44, SD = 0.56).

Additionally, the group who rated their care quality as “Good” or “Very good” also perceived the HCP to be more knowledgeable about premenstrual symptoms or disorders (mean = 3.23, SD = 0.69; U = 1370.50, *p* < 0.001) compared to those with poorer care experiences (mean = 1.60, SD = 0.72).

Moreover, individuals who rated their care quality as “Good” or “Very good” more often reported that they had received recommendations for or referrals to additional sources of help by a HCP (35.44%, *n* = 28; X^2^ = 48.251, df = 1, *p* < 0.001, Φc = 0.382) compared to those who reported poorer care experiences (5.58%, *n* = 14).

Furthermore, those who reported “Good” or “Very good” care quality had more often been provided with recommendation for lifestyle changes to manage premenstrual symptoms by their HCP (36.90%, *n* = 31; X^2^ = 7.849, df = 1, *p* = 0.005, Φc = 0.152) compared to those with a poorer care experience (21.57%, *n* = 55).

### Diagnosis of PMDD

**Fig. 2:**
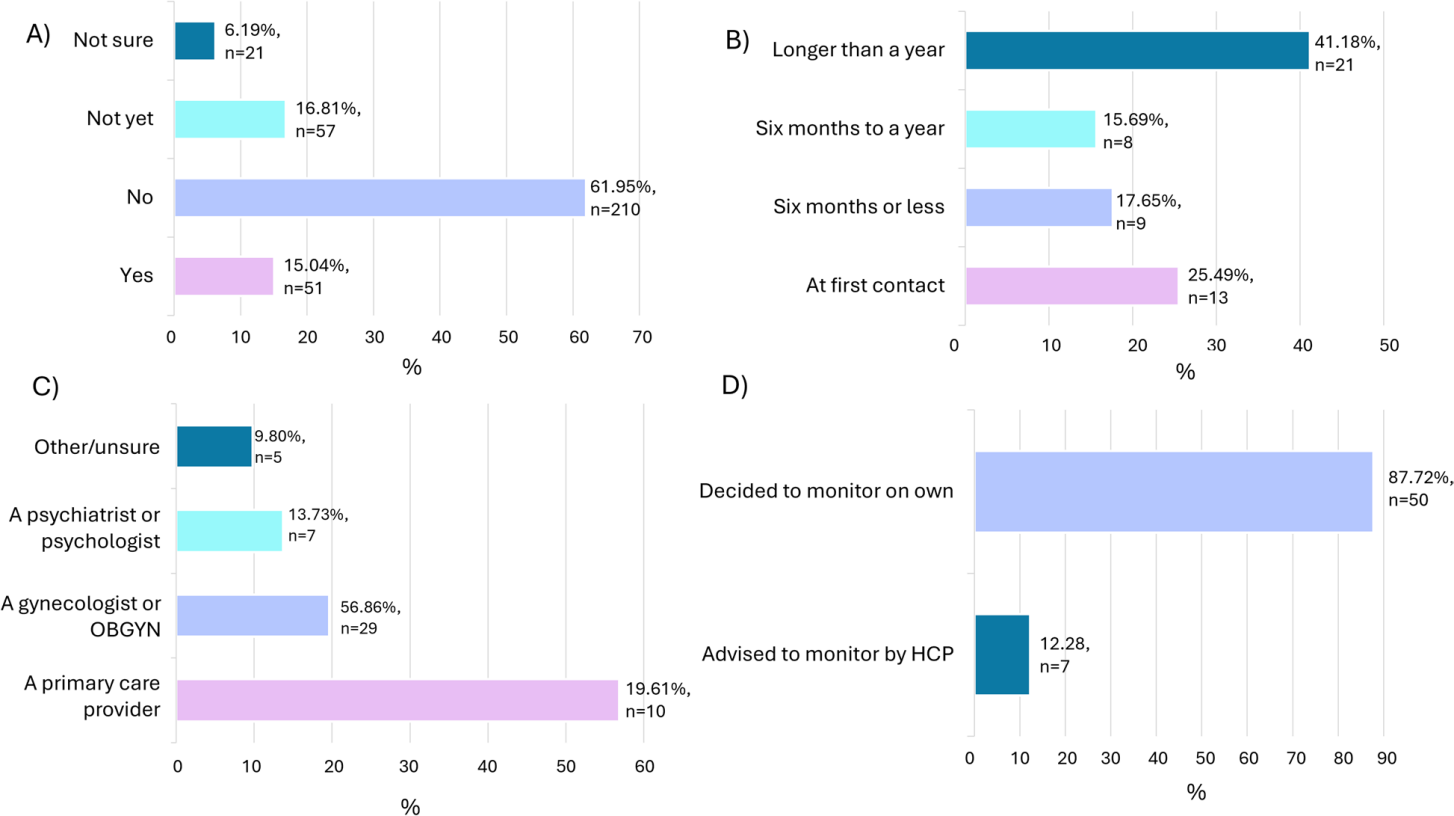
Characteristics of PMDD diagnosis. Key. **A**) Proportion of individuals who have received a formal PMDD diagnosis (*n* = 339), **B**) Time to receive a PMDD diagnosis (*n* = 51), **C**) HCP who gave PMDD diagnosis (*n* = 51), D) Initiation for premenstrual symptom monitoring (*n* = 57)

15.04% (*n* = 51) of participants reported that they have received a formal diagnosis of PMDD (Fig. 2a). Of these, the majority waited ***at least*** 12 months for a diagnosis (41.18%, *n* = 21; Fig. 2b). The majority received a formal diagnosis of PMDD from a primary care provider (56.86%, *n* = 29; Fig. 2c). 16.81% (*n* = 57) of those who sought formal help specifically for premenstrual symptoms reported currently monitoring their symptoms to assess whether they may have PMDD, with only 33.33% (*n* = 7) doing this following the advice of a HCP, with the remaining deciding to do this themselves (Fig. 2d).

### Thematic analysis

315 (92.92%) participants provided a response to the free text question regarding what could have improved their care experience. Of these, 16 (5.08%) were deemed not applicable and so removed for analysis and the remaining data (*n* = 299, 88.20% of entire sample) was included for thematic analysis. Of these 299, 20.74% (*n* = 62) characterised their overall care for premenstrual symptoms as “Good” or “Very good”, with the remaining 79.26% (*n* = 237) of participants characterising their overall care for premenstrual symptoms as “Poor” or “Very poor”. Thematic analysis revealed a total of 59 codes which were organised into 8 themes. To see all codes with code descriptions, frequencies, and illustrative quotes see Table 1.

**Table 1:** Themes, codes, code descriptions, code frequencies, and associated illustrative quotes. Total percentages may exceed 100% because multiple codes/themes can apply to a single data point. Percentages were calculated by dividing each code's frequency by the total number of responses (*n* = 299)

Theme	Code	Code description	n (%)	Illustrative quote(s)
**Empathetic care provision**	Taking symptoms seriously	Symptoms are taken seriously by the HCP(s), the patient is believed, and patient is not dismissed	75 (25.08)	“A health care professional that took my concerns seriously and didn't outright dismiss them”
	Supportive care	Acting in a way which is perceived as supportive or understanding	65 (21.74)	“At least an attempt at empathy and understanding”
	Active listening	Patient feels listened to by the HCP(s)	53 (17.73)	“Being sincerely listened to”
	Proactive approach	HCP(s) perceived as being proactive in investigating symptoms and finding an appropriate treatment/management intervention	23 (7.69)	“…wanting to find a way to help/solution/ cause” “Some curiosity.”
	HCP(s) accountability	HCP(s) provides an explanation of why they are unable to provide effective care	2 (0.67)	“A better explanation of why they couldn't help with the issues”
	Reduce feelings of burden-someness	Reduce the feeling of being perceived as a burden or nuisance by the HCP(s)	1 (0.33)	“I just felt like a nuisance”
**HCP(s) education, understanding, & research**	Improved HCP(s) knowledge and education	Improved HCP(s) education and knowledge of premenstrual symptoms and disorders (including symptoms, potential investigations, management/treatment options)	61 (20.40)	“Professionals being more knowledgeable about PMS/PMDD.”
	Recognition of impacts on functioning	HCP(s) recognising and validating the functioning impacts (e.g., workplace, relationships) that premenstrual symptoms have	15 (5.02)	“Some understanding of the impact that PMS can have, the danger of regularly feeling suicidal and the strength of symptoms.”
	Recognition of menstrual cycle role	HCP(s) not recognising the role of menstrual cycle in premenstrual symptoms and in other mental health symptoms/conditions	13 (4.35)	“Them acknowledging the possibility of period related issues”
	Research need and awareness	HCP(s) awareness of up-to-date or ongoing research, or a general need for more research into premenstrual symptoms and disorders (e.g., causes of premenstrual symptoms, treatments for premenstrual symptoms)	8 (2.68)	“Being able to speak to a practitioner with specialist knowledge and interest in PMS and PMDD, who was more up to date with the latest knowledge and treatments.”
	HCP(s) experience	Requirements/preference to see HCP(s) with experience of treating premenstrual symptoms	7 (2.34)	“Having talked with a professional who is expert on PMS” “It was like they’d [the HCP(s)] had no previous experience with the condition”
	Improved education of hormone medications	Improved HCP(s) education and knowledge of the range of benefits or risks (including potential side effects) associated with hormonal medications	7 (2.34)	“More understanding of hormonal contraception on mental health”
	Perceived focus on weight	Perception that HCP(s) are overly focused on the weight of the individual, with this impacting their symptom assessment or the treatment being offered	2 (0.67)	“Being treated as a person and having my symptoms discussed as they are unrelated to my weight.”
	Recognition of impacts on neurodevelopmental conditions	Recognition of the role of the menstrual cycle and premenstrual symptoms/disorder in individuals with neurodevelopmental conditions (i.e., autism spectrum disorder)	2 (0.67)	“…more knowledge and education on PMDD and how it affects people especially those who are neurodivergent like myself as [it] affects every aspect of our being and destroys our lives.”
	Communication with specific patient groups	Improved HCP(s) awareness of and communication with patients from specific groups or demographics (e.g., patients with disabilities, LGBTQIA + patients)	2 (0.67)	“Better disability and LGBT awareness among staff.”
	Awareness of complex symptom management	HCP(s) awareness of how to manage complex or atypical symptom presentations	1 (0.33)	“being willing to […] work with more complex cases that don't necessarily fit the flowchart.”
**Comprehensive symptom assessment and investigations**	Comprehensive symptom assessment	Asking about a wide range of possible symptoms to ensure assessment is comprehensive	28 (9.36)	“…a holistic assessment of my symptoms” “Asking more questions rather than writing it all off as ‘normal’.”
	HCP(s) offers tests/investigations	Providing tests/investigations (e.g., blood tests, scans) to rule out other underlying health problems of premenstrual symptoms	20 (6.69)	“…maybe a suggestion of a blood test to make sure hormonally everything is ok.”
	Utilization of care record/medical history	HCP(s)s using the care record/medical history during appointments to guide the appointment structure or to reduce/remove the need for repeated information gathering	9 (3.01)	“Checking my medical records prior to or in my appointment, which would have shown a PMDD diagnosis, that I was in private talking therapy and that I was taking sertraline at a consistent dose. I had to tell the GP all of this so her suggestions weren't helpful or understanding.”
	Improved speed of understanding	Earlier recognition of symptoms or understanding of experience by HCP(s)	4 (1.34)	“It took a long time/a few doctors for anyone to even mention PMDD”
	Structured screening	Using a structured symptom questionnaire during assessment	2 (0.67)	“There needs to be a new checklist for females with symptoms mental and physical with regards to periods.”
	Asking about suicidality/self-harm	HCP(s) specifically asks about/assesses suicidality/self-harm to prompt people to disclose who otherwise may be hesitant to do so	2 (0.67)	“Asking the right questions so patient doesn't have to be the one to bridge topic of SI/SH [suicidal ideation/self-harm]—it's understood that that can be apart of it”
	Recommendation of symptom tracking/monitoring by HCP(s)	HCP(s) recommends that the patient tracks/monitors their premenstrual symptoms over multiple/consecutive menstrual cycles	2 (0.67)	“It would have been better if the GP […] asked me to continue to register all my symptoms”
	Discuss test results	Opportunity to discuss results of test/investigations	2 (0.67)	“Follow up on test results, even though in normal range and discussion about what to consider next outside of basic tests”
	HCP(s) focus on physical	HCP(s) on focused on physical premenstrual symptoms/pain	2 (0.67)	“More exploration of PMS symptoms rather than the bleeding itself”
	HCP(s) identify changes in symptoms and behaviour over time	HCP(s)s should monitor symptoms and behaviours over time, in the context of the patient, to identify deterioration	1 (0.33)	“The previous GP could have looked into my symptoms better since it was a severe and unusual behavioural change, […] and noticed that this was not ‘attention seeking behaviour’”
**Diagnosis**	Provisional/ official diagnosis	Providing a provisional or official diagnosis	10 (3.34)	“Help with a diagnosis.” “having a working diagnosis”
	Symptom misattribution/ misdiagnosis	Misattribution of symptoms to another disorder (e.g., personality disorder) or misdiagnosis with another disorder	5 (1.67)	“…often blamed on stress when women go seek help for it.” “Not being told I was ‘depressed’ which, for years, was a catch-all for my mental health.”
	Support before diagnosis	Access to treatment/support without needing a diagnosis	1 (0.33)	“Support for the physical and mental health symptoms, even in lieu of a diagnosis
**Professional support and treatment provision**	Wider treatment offering from HCP(s)	Offering alternative or a range of treatment options, including hormonal, non-hormonal, and psychological treatment options	42 (14.05)	“I would also like more non medication options for treatment and support.”
	Providing treatment review	Offering follow up appointments where treatment can be reviewed and optimized	19 (6.35)	“A follow up appointment booked in at the time of starting a month of treatment to be able to check in with the doctor”
	Perceived inappropriate prescribing	Perceived that the HCP(s) prescribed inappropriate medication (based on later treatment response to different medication, own research, etc.)	8 (2.68)	“I don’t think it’s appropriate to prescribe SSRIs for PMDD, when said SSRIs also come with unbearable side effects”
	Negative impacts of medication	Not wanting a specific medication/ treatment due to previous negative experience of medication for premenstrual symptoms/ disorders (e.g., hormonal medications, antidepressants) such as side effects	8 (2.68)	“The feeling that if I do seek help it could be resolved without going back into contraceptives as they ruin my skin and give me vaginal dryness, lack of overall sex drive.”
	HCP(s) providing treatment	HCP(s) making a care plan and providing treatment for premenstrual symptoms/disorders	6 (2.01)	“Offering real help, trying treatments, actually trying to solve the problem.”
	"one size fits all"	Recognising the individual and delivering personalized care and treatment management, no "one size fits all" approach	4 (1.34)	“…not given a one size fits all answer” “…individual treatment. It felt like they just have a one size fits all answer which doesn't work”
	Improved speed of treatment initiation	HCP(s) providing a treatment at first instance of help-seeking	3 (1.00)	“Counselling should be absolutely offered straight away” “If they had prescribed [the contraceptive pill] straight away”
	Treatment uncertainty	Uncertainty about available treatment/management options	2 (0.67)	“I'm not sure what other methods of support there are as the GP didn't speak about any—does the contraceptive pill help?”
	Ensure medication continuity	Providing continuity of medication (i.e., ensuring that access to hormonal medication is maintained)	1 (0.33)	“My GP […] hasn't always understood the importance of me always having access to the pill (despite it being written on my record it's for PMDD)—I can't just wait like everyone else or the symptoms return.”
	Support in securing time off work	Support from HCP(s) in securing time off work	1 (0.33)	“Discussion of PMDD and how it could allow time off work if necessary.”
**Signposting or referral to additional resources or sources of help**	HCP(s) providing additional information	HCP(s) providing in-appointment information or recommendations of or referrals to other sources of information about premenstrual symptoms and disorders (including symptoms, treatment/management options)	48 (16.05)	“It would have been better if the GP explained my symptoms to me and explained what is happening to me” “I was never made aware of any PMDD-specific resources.”
	HCP(s) providing advice	HCP(s) providing advice on how to manage symptoms through lifestyle changes	35 (11.71)	“For advice and guidance related specifically to the menstrual cycle—no medical professional has ever spoken to me about this without me specifically bringing it up.”
	HCP(s) providing an onward referral	HCP(s) referring the patient to a relevant medical specialist (e.g., a gynaecologist)	31 (10.37)	“…to refer people to ACTUAL[ly] get help to treat their problems”
	HCP(s) signposting to sources of support	HCP(s) providing recommendations of or referrals to other sources of support (e.g., support groups/services)	15 (5.02)	“it might have been useful to be directed to other sources of support.”
	Information on symptom frequency	More information provided regarding how common symptoms are in women and people assigned female at birth	2 (0.67)	“…what range of symptoms are commonly experienced”
	Request for “patient packs”	Request for patient packs (i.e., with information, additional sources of help) to be provided by HCP(s)	1 (0.33)	“Patient care packs—daily diary, helpline numbers and self-help book”
**Wider healthcare system improvements**	Decreased wait time	The speed of an onward referral or time between appointments decreasing	9 (3.01)	“…it was the referral to the psychiatrist and gynecologist when things slowed down/stopped”
	Longer appointment time	Requirement or preference for a longer appointment with HCP(s)	8 (2.68)	“When I began to list the different symptoms I was told I'd need to book separate appointments to discuss them”
	Improve HCP(s) access	Making it easier to make an appointment with or contact HCP(s)	6 (2.01)	“It was near-impossible to get a doctor's appointment at my surgery, so more availability of appointments would have helped.”
	HCP(s) continuity	Continuity of the same HCP(s) across appointments	3 (1.00)	“Continuity of care—after I was referred for blood tests and they came back clear, there was no follow-up”
	Specific treatment pathways	Specific treatment pathway for premenstrual symptoms/disorders	2 (0.67)	“A clear referral pathway to get a diagnosis”
	Self-referral to specialist care	Available pathways to enable self-referral to specialist services	1 (0.33)	“Ability to self-refer to [obstetrics-gynaecology]/consultant services.”
	Transparent referral decision making	Explanation of why referrals to specialist services have not been accepted	1 (0.33)	“The [gynaecology] department taking the initial referral […] instead of pinging it back to my GP with no real reasoning as to why.”
**Patient role/voice and preferences**	Preference for face-to-face appointments	Request for more opportunity for or preference for face-to-face conversation/appointment	8 (2.68)	“Face to face where I felt I was actually listened to”
	Encouraging shared care decision making	Perceived lack of shared decision making	6 (2.01)	“Not been defensive when I questioned whether antidepressants were the best option.”
	Respect treatment decisions	HCP(s) respecting the patient's decision not to use certain treatments	5 (1.67)	“Listened to my concerns about using anti-depressants and worked with me to discuss alternative solutions.”
	Preference for female HCP(s)	Preference expressed to be seen by a female HCP(s)	4 (1.34)	“Maybe having a female practitioner to speak to instead of a male”
	Consider patient gathered information	HCP(s) takes information patient has gathered from informal sources seriously (e.g., from online)	3 (1.00)	“Also not rolling their eyes when I’ve said I’ve looked on the internet when I’ve done that as I’ve felt unheard by the very people who should be listening”
	Safety plan	Co-design of safety plan between HCP(s) and patient	1 (0.33)	“Next of kin/ trusted person patient and GP should do a safety plan also what can those do to help and keep patient safe.”
	Ability to provide information prior to appointment	Ability to contact HCP(s) prior to appointment (e.g., via email or online form) to provide details of symptoms and their impact to guide appointment	1 (0.33)	“Before when I had my first appointment, I sent in an email with all my symptoms so the Dr could read before I went in. That helped because I felt he guided the conversation and I kind of knew what we would discuss.”

### Empathetic care provision

Empathetic care provision was identified as a theme (Table 1), with participants stating the importance of HCPs taking premenstrual symptoms seriously (e.g., “*They could have at least made me feel as though they took me and my symptoms serious[ly]”)* and acting in a way which was perceived as supportive (e.g*., “Being more compassionate, more understanding”*). They also emphasised the importance of HCPs engaging in active listening (e.g., *“Please listen to women! We don’t come to the GP for fun. We are usually embarrassed and desperate by the time we seek help so please don’t fob us off”*). Active listening was often associated with a proactive approach, where the HCP is perceived as being proactive in care. This may include offering suggestions of other sources of support without the patient needing to ask (e.g., *“For advice and guidance related specifically to the menstrual cycle—no medical professional has ever spoken to me about this without me specifically bringing it up”*), or taking the time to do research and expand their knowledge if they were unfamiliar with premenstrual symptoms or disorders (e.g., “*If he had said he didn’t know much about it but would look into it for me and come back to me with what he’d found so he could help me”*). Crucially, participants raised concerns about the potential risks associated with empathetic care not being delivered, particularly if help-seeking occurs in the luteal phase (e.g., “*If they had spoken to me as disrespectfully and dismissively on that week when I was extremely vulnerable, I dread to think the outcome.”).*

### HCP(s) education, understanding, and research

Many participants mentioned the importance of improved HCP knowledge of premenstrual symptoms and disorders (Table 1), including potential symptoms and their treatment/management (e.g*., “If my GP had had knowledge of PMDD in any form this would have helped.”*). The improvements in HCP knowledge called for by participants included improved recognition of the potential impacts of premenstrual symptoms and disorders on functioning (e.g., *“…recognising the impact it has on my life which has been practically ruined.”*). Additionally, some participants stated there was a need to more generally improve recognition of the role of the menstrual cycle in mood and mental health (e.g., *“Assumptions were made by a male doctor who put it down to depression. He had no intention of listening to the fact that just before my period I had thoughts of suicide but it was instantly relieved once my period has started.”*).

There was also a sentiment that there was a need for further research into premenstrual symptoms and disorders, including improved understanding of the causes and presentation (e.g., *“More research and understanding about PMS—what range of symptoms are commonly experienced and for how long, what is considered usual, do hormone levels play a role and can we consider testing levels at different times if the cycle to see if there is any dysregulation that is causing extreme PMS?”*) as well as more evidence for the efficacy of available treatment/management options, in addition to the discovery of novel treatment/management options (e.g., *“More research into what causes these issues and an effective treatment that deals with the acute problem rather than blanket long term medications for symptoms.”*). Participants also remarked that it is important that HCPs are aware of recent research to inform high-quality care practices (e.g., *“Further knowledge and understanding of up to date research into appropriate care for PMDD”*).

### Comprehensive symptom assessment and investigations

Participants raised the importance of comprehensive symptom assessment, which asks about a wide range of possible premenstrual symptoms (Table 1). Some patients commented that comprehensive symptom assessment may be achieved by employing a structured screening tool to ensure all premenstrual symptoms are covered (e.g., *“Maybe going through a questionnaire to guide questions about relevant symptoms”*) as this would also ensure high-risk symptoms and behaviours such as suicidality and self-harm are assessed, with many participants reporting that their HCP(s) had not specifically asked about suicidality and self-harm (e.g., *“Asking the right questions so patient doesn't have to be the one to bridge topic of [suicidal ideation/self-harm]—it's understood that that can be a part of it”*). This is especially crucial as some patients may not feel able to disclose this information without prompting from a HCP (e.g., *“I was too scared when my children were younger to admit that I felt suicidal every month.”*).

As part of a comprehensive symptom assessment, some participants expressed wanting to have tests/investigations such as blood tests or scans performed to rule out any underlying causes and identify potential hormonal imbalances (e.g., *“If she has taken me seriously, done further tests, or even pretended to care.”*) or a complete psychiatric assessment that considered other mental health or neurodevelopmental conditions (e.g., *“I would also have liked more conversations about ruling out other issues and investigating comorbidities like ADHD”*). However, many participants also noted that a crucial aspect of offering tests/investigations is ensuring there is an opportunity to discuss results from these tests/investigations with a HCP (e.g., *“Follow up on test results, even though in normal range and discussion about what to consider next outside of basic tests”*).

As a further aspect of delivering a comprehensive symptom assessment, some participants also suggested that the care record or patients’ medical history could be a useful supplement to appointments to contribute to a comprehensive assessment by streamlining and guiding the appointment (e.g., “*If GPs who had looked through my history prior to calling and understood my situation had been more thoughtful and treated me with the respect I needed at the time.”*) Using the care record would also reduce or remove the need for repeated information gathering (e.g., *“…glancing through my history while we spoke so I wasn't going over the same points again and again each time*”) as well as facilitating follow-up and monitoring over time, and between HCP(s) (e.g., *“…compare notes over my years & diff NHS [departments]”)*.

## Diagnosis

Participants expressed wanting to have received a diagnosis of PMDD, even if this was provisional (i.e., whilst tracking symptoms over two cycles) or “A formal diagnosis” (e.g., *“A proper diagnosis instead of just being brushed off with ‘you are a woman that is just normal’”*). Participants also reported concerns of misattribution of their premenstrual symptoms to another cause such as stress and concerns of misdiagnosis of another mental health condition such as depression (Table 1).

### Professional support and treatment provision

The theme of professional support and treatment provision (Table 1) was mainly characterised by participants expressing the need of a wider treatment offering from HCPs, this includes offering alternative or a range of treatment options including hormonal, non-hormonal, and psychological treatments (e.g., “*Improved variety of suggestions to help treat or manage symptoms.”*). This code also reflects a perception that only hormonal (e.g., *“Not being palmed off with the standard contraceptive options”)* or antidepressant (e.g., *“Resources being available for more support options—not just antidepressants.”)* treatments will be offered by HCP(s). Related to this, some participants reported a general sense of uncertainty about available treatment/management options (e.g., *“Clearer management/treatment options.”*).

There was a perception from some participants that they had received an inappropriate prescription (e.g., *“Not been prescribed pain medication that I cannot take, and could have caused me more harm than good if I hadn't checked the label myself.”*) Associated with this, many participants having had previous negative experiences with medication available for management of premenstrual symptoms and disorders (e.g., “*Hormonal treatments are the first and apparently only treatment for anything related to your cycle, I felt pushed towards that as the ‘best’ option even though I’ve had such bad experiences with them in the past.”*). In some cases this negative treatment experience resulted in reduced interest in further treatment seeking behaviour (e.g., *“But realistically other than medication to mask the symptoms of PMDD at the expense of worse side effects, there is nothing they can do for me.*”) or reported treatment non-adherence (e.g., *“I stopped taking [the contraceptive pill] because it was giving me so many side effects.*”). This also highlights the importance raised by participants of HCPs providing a treatment follow-up to ensure treatment efficacy and optimisation, as well as monitoring negative side-effects (e.g., *“When put on the pill (especially after I told them it causes me to have low moods), a follow up of how I was finding it after 3 months.”*).

### Signposting or referral to additional resources or sources of help

Participants generally requested the provision of additional information from HCPs (Table 1), either provided directly by the HCPs in an appointment setting or the HCPs signposting to resources which can be independently accessed outside an appointment setting (e.g., “*I wasn't signposted to any support networks or relevant information.”*). Some participants specifically mentioned that this information should include information about the common types of premenstrual symptoms experienced which would allow them to compare their experiences against others (e.g., *“I would have wanted more understanding of how widespread the symptoms are, how common among women they are, so I didn’t feel so alone.”*) Participants also stated that receiving advice for symptom management from HCPs would be welcome (e.g., “*Some advice on what could help during these two weeks.”*). Many participants also mentioned wanting the HCPs to provide an onward referral to specialist care (e.g., *“Let me see a gynecologist, someone specialized.”*) or to signpost to other sources of help (e.g., *“…being signposted to charities or other specialized professionals”*).

### Wider healthcare system improvements

Some participants commented on factors which would improve the care experience which are relevant to wider healthcare system improvements (Table 1). One such improvement is decreasing wait times, this included both reducing the speed of an onward referral (e.g., *“Referral could be quicker”*) and the time between appointments decreasing (e.g., *“Improved waiting times between appointments.”*). Related to this, participants noted that it was important for access to HCPs to be improved (e.g*., “Being easily able to access GP (or other appropriate primary care) for a 'non urgent' matter.”*). It was also mentioned that continuity of HCPs between appointments was crucial, with a single HCP acting as a single point-of-contact during the diagnostic and treatment process (e.g., *“…to have the same female GP as a point of contact.”*). Participants also noted it would be beneficial if appointment times were longer (e.g., *“More time for appointments to really go through information. They often seem rushed.”*).

### Patient role/voice and preferences

The patient role/voice and preferences theme (Table 1) was mainly characterised by a preference for face-to-face appointments with HCP (e.g., *“More dialogue, face to face appointment”*). With a further preference reported by some participants to see a female HCP (e.g., *“Seeing a female consultant*”). Participants also expressed interest in engaging in their healthcare experiences and highlighted the importance of the HCPs encouraging shared decision making with the patient, particularly in regards to treatment (e.g., “*A dialogue about treatment options.”*), with it additionally being crucial for the HCPs to respect the patient’s treatment decision (e.g., *“More patience from the doctor, and respect for my choice not to want to get on the pill.”*). Participants also stated it may be useful and increase patient involvement if the HCPs considered information gathered by the patient prior to the appointment from informal sources (e.g., from online resources) (e.g., *“Sometimes I feel like having done some research is off putting to general practitioners and I have to play stupid in order to be advised. If I come in with some theory as to what may be the problem, I often get a bad look or the tone of the conversation completely changes (for the worse).*”).

## Discussion

The current study aimed to examine care experiences for premenstrual symptoms and disorders in the UK, while also seeking to identify potential strategies for care improvements. The novelty of this study lies in its exploration of healthcare experiences of individuals seeking care for premenstrual symptoms, regardless of a formal diagnosis. This accounts for the perspectives of individuals who may be undiagnosed, misdiagnosed, or awaiting a diagnosis, as well as those who do not meet the clinical threshold but continue to experience impactful symptoms that they feel warrant professional support. By not limiting the sample to individuals with a formal diagnosis, this study offers recommendations for improving care that could be more broadly applicable across the full spectrum of potential of formal help-seekers, including those with milder symptoms. Additionally, by capturing the narratives of those who have sought help for premenstrual symptoms, we aim to identify potential areas for improvement from their personal experiences. This approach places the perspectives of this group at the forefront, aligning with recommendations from the Women’s Health Strategy 2022 [20].

The study reflects previous findings [16], also demonstrating broadly poor care experiences for premenstrual symptoms. The sample generally perceived that HCPs held dismissive attitudes or acted dismissively. One possible explanation of this perceived dismissiveness is that HCPs may view premenstrual symptoms as a common, potentially clinically irrelevant part of the menstrual cycle, especially in the absence of a PMDD diagnosis. This could result in an underestimation or dismissal of these symptoms and their potential impact. However, it is important to highlight that even individuals without a formal PMDD diagnosis can experience significant distress from premenstrual symptoms, which may still warrant formal support. Therefore, it is crucial to recognise that the absence of a PMDD diagnosis does not diminish the validity or impact of premenstrual symptoms. There was a widely held perception that HCPs also lacked knowledge of premenstrual symptoms and disorders, with this mirroring the perception of HCP knowledge of women’s health more generally [20]. Healthcare professionals may need more comprehensive training to distinguish between normal premenstrual experiences and those that significantly affect quality of life. In fact, HCPs report frustration with the lack of training they receive in respect to premenstrual disorders [21]. Improving training would ensure that individuals experiencing distressing symptoms, regardless of a formal diagnosis, receive the appropriate attention and support, even if this is suggestions of lifestyle changes or signposting to other support options outside of the clinical setting. However, the majority of the sample had not received advice on potential lifestyle changes which could be made to manage symptoms, despite this being a first line treatment recommendation [9, 10]. Further, such information would be a highly valuable resource as education on available lifestyle changes can empower individuals to self-manage premenstrual symptoms [22], which could be leveraged for therapeutic benefits either outside of or alongside other interventions. Not receiving these recommendations directly from a HCP would likely necessitate individuals to find this information independently. With the majority of the sample also not receiving recommendations of or referrals to other sources of information from a HCP, the necessity of independent searching is further increased, with there being a risk of relying on poor quality information [23]. While awareness of these options may be gained independently of HCP involvement, if a HCP is consulted, care experiences which are perceived as dismissive should not be the norm, even when symptoms do not meet the diagnostic threshold.

There was also an apparent delay to diagnosis of PMDD in the current sample, with the majority of those with a PMDD diagnosis reporting a wait of at least a year. Delays to diagnosis of up to 14 years are reported for PMDD [21], with individuals often having to consult multiple HCPs with no HCP continuity [13] as also reported by participants in the current study. Delays to diagnosis of premenstrual disorders are associated with considerable challenges for the individual. Notably, previous qualitative research has revealed the high levels of emotional distress, reliance on maladaptive coping mechanisms, and unstable self-image experienced while awaiting a formal diagnosis of PMDD [16]. In contrast receiving a diagnosis results in a considerable sense of relief for the individual, providing an explanation for their symptoms [13]. Therefore, reducing delays to diagnosis is crucial to improve quality of life and reduce suffering. Interestingly however, the receipt of a provisional or official diagnosis was not frequently mentioned in the qualitative data as important for improving healthcare experiences. Instead, the qualitative data suggests that comprehensive symptom assessment is more frequently considered important for high-quality care experiences. In the current study GPs provided the majority of diagnoses of PMDD and so their ability to accurately identify symptoms when first consulted is critical. One approach to achieve this is through training for HCPs specifically on premenstrual symptoms and disorders, with a focus on symptom recognition. Another avenue for improved symptom identification is using a validated screening tool when patients present with a complaint of premenstrual symptoms, with qualitative data also demonstrating interest in such a tool from a patient perspective. Whilst prospective tracking of symptoms across two menstrual cycles is required to confirm a formal diagnosis of PMDD, screening would provide a provisional diagnosis [24] and identify individuals who require further assessment [25]. Comprehensive mental health screening could also be used to rule out other conditions with an overlapping symptom profile such as major depressive disorder [26], and to initially establish whether symptoms exclusively occur in the luteal phase. Perhaps crucially, use of a validated and standardised tool for the assessment of premenstrual symptoms in a clinical setting would also ensure that the HCP as standard practice asks about high-risk psychological symptoms which some participants reported difficulty in disclosing.

Whilst these findings paint a disappointing picture of the current care provision for premenstrual symptoms in the UK, they should not be interpreted as criticism of HCPs working in the UK who are involved in the management of such symptoms. Instead, we intend them to be used as a call to action, highlighting the need to make improvements to healthcare experiences for premenstrual symptoms and disorders. As such, the current study also revealed factors which underpin more positive care experiences. Some participants raised suggestions of wider healthcare system improvements which they perceived to be required for better healthcare experiences. Core suggestions included faster referrals, ensuring continuity of care with a single point of contact between appointments, and longer appointment times. Whilst these are likely to be important for delivery of high-quality care, they are not easily addressable, especially considering the current strains experienced by the national health service in the UK [27]. However, other findings from the current study may be more feasible to enact for service improvement.

Firstly, participants who perceived their HCP as knowledgeable reported more positive care experiences, with qualitative data also indicating that consultation with a knowledgeable clinician would confer a better experience. In the current study, primary care providers were the most frequently, and in many cases the only HCP consulted and so are likely the sole source of formal management of premenstrual symptoms. For this reason, in the UK, GPs have been highlighted as being the prime candidates for improved education in women’s health [20]. Unfortunately, research has shown that globally primary care providers are the medical specialty perceived as the least knowledgeable about PMDD [28]. Going forward, the UK government has pledged to improve the provision of training in women’s health for incoming doctors [20, 29]. However, this does not address potential lack in knowledge in currently practicing HCPs. Although specific training courses are available for HCPs, they are not mandatory and therefore uptake is likely limited to those clinicians with a special interest. Therefore, although we encourage currently practicing HCPs to engage with available training for premenstrual symptoms and disorders, an alternative is providing clinicians with “toolkits”. These toolkits could offer key information for identification and management of premenstrual symptoms and disorders. Toolkits have demonstrated some promise in communicating health information and research evidence in healthcare [30, 31], but further research is required to fully determine the impacts of implementation [30, 31]. Toolkits are perceived as particularly effective by clinicians and other allied health professionals when they are actionable, concise, and have a flexible delivery modality [32]. It is also vital that toolkits have a clear purpose, strong-evidence base, and are subject to evaluation in the setting of interest [31]. Therefore, future research may wish to explore the development of toolkits for premenstrual symptoms and disorders. The primary intended benefit of such tools would be to improve HCP knowledge, with a secondary intended benefit of improving care experiences. In future, if such toolkits for premenstrual symptoms and disorder are integrated into care settings to support HCPs in delivery of care, monitoring uptake and impact will be crucial.

Secondly, attentive attitudes from HCPs were associated with better care experiences. The qualitative data also indicated the importance of supportive care for positive care experiences, with HCP active listening and taking symptoms seriously deemed to be key. This may be addressed by offering training to healthcare professionals to improve patient-centred communication skills [20, 33]. Patient-centred communication uses varied strategies including active listening and use of layperson language in order to build a strong therapeutic relationship, gather and provide relevant information, and facilitate shared decision-making [34]. Taken together, this can improve the overall care experience and outcomes [34]. However, some clinicians report trepidation about utilising patient-centred communication due to time constraints during consultations [34]. Given that GPs in the UK spend an average of under 10 minutes in consultation with patients [35], this is likely a restriction for comprehensive symptom assessment and creation of personalised care plans. Previous literature has also recommended that dismissive care interactions may be improved by training patients in effective health communication, with a focus on how to effectively ask for a preferred intervention [33], with participants also noting the importance of the HCP enabling opportunities for shared decision making. However, whilst this approach may empower the patient, it again places the burden of communication on them. Instead, a mixture of patient-centred communication and self-advocacy should be used to establish the patient’s circumstances, symptoms, needs, and preferences. Doing so will likely result not only in better care experiences, but also improved overall outcomes.

Further, as recommendations of additional information sources and lifestyle changes from the HCP were found to confer a better care experience from both quantitative and qualitative data, it is vital that HCPs are empowered to provide these recommendations. Whilst improved training and toolkits can provide an increased knowledge of relevant recommendations, an additional offering may be a database of high-quality websites and support groups. This database would enable personalisation of recommendations for additional information or support based on patient-specific factors. In addition to general information and advice, qualitative data revealed the perception that there would be, or previous experience of, a limited treatment offering from HCPs. This highlighted the importance of improved information provision about the full range of treatments available for premenstrual symptoms and disorders. Such information would likely reduce hesitancy in treatment-seeking by addressing uncertainty around the availability and appropriateness of treatment options, whilst also increasing trust that the patient is able to refuse specific treatments in favour of others. It also further demonstrates the importance of improved HCP education to ensure they are aware of all therapeutic interventions available so they can offer comprehensive treatment options and enable the patient to be involved in the decision-making process. This also underscores the importance of amplifying patient voice and ensuring they are included in a participatory role in their care, which was deemed important for positive care experiences. Further, it was deemed highly important that treatment reviews were offered to monitor the treatment response and safety, as well as optimising the therapeutic intervention as required.

## Limitations

The results of the current study should be considered in the context of several limitations. Firstly, the survey questions were not formally reviewed or evaluated by patient representatives. While the survey was reviewed by an experienced consultant psychiatrist, input from a broader range of healthcare professionals and patients could have strengthened the validity of the survey. Future research would benefit from incorporating feedback from these groups to ensure the survey captures relevant perspectives on care experiences.

Secondly, there could have been a recruitment bias due to the sole use of social media for recruitment, which may have limited participation to individuals who are frequent social media users and who may not be fully representative of the broader population. Additionally, recruitment materials specified a focus on healthcare experiences for premenstrual symptoms, which may have motivated individuals with particularly positive or negative experiences to participate. The recruitment materials are also likely to have attracted participants who are more informed or educated about premenstrual symptoms, and thus may not capture the experiences of those with less awareness or those who do not actively seek care for their symptoms. Further, the majority of the sample were white and identified as women. Therefore, the results will not reflect the healthcare experiences of individuals from minority or other demographic groups who are likely to already experience poorer care [36] or be more easily dismissed by HCPs [33]. Finally, by excluding individuals diagnosed with other gynaecological disorders or those who no longer experience premenstrual symptoms, the results may be biased toward more recent or negative experiences of care, potentially overlooking positive outcomes from effective care or treatments. Therefore, additional research is needed in a more diverse sample to explore the quality of care for premenstrual symptoms more broadly, particularly to understand the experiences for individuals who may face additional barriers to receiving high-quality care. In future, incorporating additional recruitment methods beyond social media such as through healthcare settings, community organisations, or offline advertisements may be beneficial for reducing the risk of recruitment bias by reaching a wider range of individuals.

Additionally, it is worth considering that some scales lacked neutrality and that the placement of open-text questions after closed questions may have inadvertently shaped participants' responses, and potentially resulted in the reporting of more negative care experiences.

Finally, there are likely to be other determinants of positive healthcare experiences for premenstrual symptoms and disorders which were not considered in the current study. Previous research investigating factors influencing perceived quality of care identified a range of structural, interpersonal, and patient-level determinants [37, 38]. Therefore, whilst the current study reveals some factors, future research is required to identify further determinants of high-quality care.

## Conclusions

To conclude, poor care experiences for premenstrual symptoms are pervasive in the UK characterised by dismissive attitudes and perceived lack of HCP knowledge of premenstrual symptoms and disorders. Thematic analysis revealed eight themes to improve healthcare experiences for premenstrual disorders: Empathetic care provision; HCP education, understanding, and research; Comprehensive symptom assessment and investigations; Diagnosis; Professional support and treatment provision; Signposting or referral to additional resources or sources of help; Wider healthcare system improvements; and, Patient role/voice and preferences. Improving the provision of training for HCP in the UK is required to deliver higher quality care for premenstrual symptoms and disorders. However, given the current constraints on the healthcare system in the UK, providing HCPs with easy to access, high-quality toolkits may deliver benefits in a faster timeframe. In tandem, the utilisation of standardised screening tools for premenstrual symptoms and disorders in healthcare settings may be beneficial for delivering comprehensive assessments which includes high-risk psychological symptoms. Additionally, we encourage HCPs to engage in patient-centred communication, where feasible, to reduce the burden of self-advocacy on the help-seeking individual and improve the care experience. Further, it is crucial to improve knowledge of therapeutic interventions for both the public and HCPs to reduce barriers to treatment-seeking and enable shared decision making.

## Supplementary Information

Supplementary Material 1.

Supplementary Material 2.

## Data Availability

The datasets used and/or analyzed during the current study are available from the corresponding author on reasonable request.
