# Supplementary material for: Improving care experiences for premenstrual symptoms and disorders in the United Kingdom (UK): a mixed-methods approach
Source: BMC Health Serv Res. 2025 Jan 14;25:70. doi: 10.1186/s12913-024-12140-3 (PMC11730124; doi:10.1186/s12913-024-12140-3)
Supplement: Supplementary file 1 — Supplementary Material 1. [file 12913_2024_12140_MOESM1_ESM.docx]

**Supplementary Document A1 (Supplementary information). Participant information sheet excerpts and relevant survey questions**

**Help-seeking and treatment experiences for premenstrual symptoms, premenstrual syndrome (PMS) and premenstrual dysphoric disorder (PMDD)**

**Participant Information Sheet**

Dear Participant,

The aim of this study is to understand factors implicated in help-seeking for premenstrual symptoms, as well as, experiences of care and treatment from healthcare professionals (e.g., general practitioners, primary care physicians, family doctors, nurses, psychologists, psychiatrists) in women who have sought help for premenstrual symptoms. Moreover, we are interested in other types of help (e.g., online or from friends and family) for managing premenstrual symptoms. Additionally, we want to explore perceptions of care and treatment those with a formal diagnosis of premenstrual dysphoric disorder (PMDD) have received.

Premenstrual symptoms can include psychological (e.g., low mood or feeling down, feeling more irritable, feeling overwhelmed, feeling anxious), physical (e.g., headaches, bloating, breast tenderness) and behavioural symptoms (e.g., food cravings, eating more than usual, sleeping more than usual). These symptoms normally occur in the week leading up to a period and start to improve within a couple of days after the start of a period.

Premenstrual syndrome (PMS) and premenstrual dysphoric disorder (PMDD) are characterised by severe premenstrual symptoms which impact day-to-day life in the week leading up to a period (e.g., making it hard to work, causing problems with romantic partners or family, making it hard to look after oneself).

Before you decide to take part, it is important that you understand what it will involve. Please take your time to read the following information carefully.

You can contact a member of the research team if you have any questions.

**Can I take part?**

You should be:

- 18 years or older

- Have a strong comprehension of the English language

- Be assigned female at birth

- Experience premenstrual symptoms

- Not be pregnant or in the menopause or menopause transition

- Not diagnosed with any gynaecological conditions (e.g., endometriosis, polycystic ovary syndrome (PCOS))

You do not need to have sought help for these symptoms from a healthcare professional (i.e., a primary care provider such as a general practitioner, primary care physician, family physician, family doctor, a nurse or secondary care providers such as a psychologist, a psychiatrist, a gynaecologist or an OBGYN) to take part.

You do not need a diagnosis of or to be receiving treatment for premenstrual syndrome (PMS) or premenstrual dysphoric disorder (PMDD) to take part.

**What is involved?**

You will be asked to complete a short online survey. We are interested in hearing about your attitudes towards seeking help for premenstrual symptoms, and experiences of the care and treatment you may have received if you have sought help for premenstrual symptoms.

The survey should take approximately 10-20 minutes to complete. You don't have to complete this survey in one go. Just make sure to keep your browser window or tab open so you can return to it. Please note that, once you have started the survey, you have a week to complete it.

**Will I be paid?**

No, you will not be paid to take part in this study.

What is your age?

________________________________________________________________

How do you identify?

- Woman
- Man
- Non-binary
- Other
- Prefer not to answer

What is your ethnic group? Please note that this does not refer to nationality (e.g., American, French, English, Polish)

- White or Caucasian
- Mixed or multiple ethnic groups
- Asian (Indian, Pakistani, Bangladeshi, Chinese, or any other Asian background)
- Black, Caribbean or African
- Arab, Arab, Middle Eastern, or North African descent
- Hispanic or Latinx
- Other ethnic group
- Prefer not to answer

What is the highest level of education you have completed?

- Primary education or below (up to the age of 11)
- Lower secondary education (up to the age of 16)
- Upper secondary education (up to the age of 18)
- Undergraduate education (e.g., a Bachelor's degree)
- Postgraduate education (e.g., Master's, PhD)
- Other
- Prefer not to answer

What is your current employment status? Please tick all that apply

- Employed full-time
- Employed part-time
- Self-employed
- Homemaker
- On parental leave or taking time off to care for a family member
- Student
- Retired
- Voluntary work
- Unemployed
- Prefer not to answer

|  |
| --- |

Have you visited a healthcare professional specifically for your premenstrual symptoms?
 
This may have been a primary care provider (e.g., general practitioner, primary care physician, family physician, family doctor) or a secondary care provider (e.g., a psychologist, a gynaecologist, OBGYN, or a psychiatrist).

- Yes
- No

Display This Question:

If Have you visited a healthcare professional specifically for your premenstrual symptoms?   This ma... = Yes

|  |
| --- |

Which healthcare professionals have you visited for your premenstrual symptoms? Please select all that apply

- A primary care provider (e.g., general practitioner, primary care physician, family physician, family doctor)
- A psychologist
- A gynaecologist or OBGYN
- A psychiatrist
- Other, please specify what healthcare professional(s) __________________________________________________

Display This Question:

If Have you visited a healthcare professional specifically for your premenstrual symptoms?   This ma... = Yes

Overall, do you think your premenstrual symptoms were taken seriously by the healthcare professional(s) you saw?

- Not at all seriously
- Yes, slightly seriously
- Yes, moderately seriously
- Yes, very seriously

Display This Question:

If Have you visited a healthcare professional specifically for your premenstrual symptoms?   This ma... = Yes

Did the healthcare professional(s) you saw seem knowledgeable about PMS and PMDD?

- Not at all
- Slightly
- Moderately
- Very
- I am not sure

Display This Question:

If Have you visited a healthcare professional specifically for your premenstrual symptoms?   This ma... = Yes

Did the healthcare professional(s) you saw recommend or refer you to other places you could seek additional information about PMS and PMDD (e.g., leaflets, websites, peer support groups)?

- Yes, without me having to ask
- Yes, but I had to ask for more information
- No
- I am not sure

Display This Question:

If Have you visited a healthcare professional specifically for your premenstrual symptoms?   This ma... = Yes

|  |
| --- |

Overall, how would you rate the quality of care you have received from a healthcare professional(s) for your premenstrual symptoms?

- Very poor
- Poor
- Good
- Very good

Display This Question:

If Have you visited a healthcare professional specifically for your premenstrual symptoms?   This ma... = Yes

In your opinion, what could have improved your care experience?

________________________________________________________________

________________________________________________________________

________________________________________________________________

________________________________________________________________

________________________________________________________________

Display This Question:

If Have you visited a healthcare professional specifically for your premenstrual symptoms?   This ma... = Yes

Have you received a formal diagnosis of premenstrual dysphoric disorder (PMDD) from a healthcare professional?

This healthcare professional could have been a primary care provider (e.g., general practitioner, primary care physician, family physician, family doctor) or a secondary care provider (e.g., a psychologist, a gynaecologist, OBGYN, or a psychiatrist).

- Yes
- No
- Not yet, but I am currently monitoring my PMS symptoms to assess whether I may have PMDD
- I am not sure

Display This Question:

If Have you received a formal diagnosis of premenstrual dysphoric disorder (PMDD) from a healthcare... = Not yet, but I am currently monitoring my PMS symptoms to assess whether I may have PMDD

You mentioned you are monitoring your PMS symptoms to assess whether you may have PMDD. Is this on the advice of a healthcare professional?

- Yes, a healthcare professional suggested or advised me to do this
- No, I decided to do this myself

Display This Question:

If Have you received a formal diagnosis of premenstrual dysphoric disorder (PMDD) from a healthcare... = Yes

|  |
| --- |

Which healthcare professional formally diagnosed you with PMDD?

- A primary care provider (e.g., general practitioner, primary care physician, family physician, family doctor)
- A gynaecologist or OBGYN
- A psychologist
- A psychiatrist
- Other, please specify __________________________________________________
- I am not sure

Display This Question:

If Have you received a formal diagnosis of premenstrual dysphoric disorder (PMDD) from a healthcare... = Yes

|  |
| --- |

Approximately, how long did it take for you to receive a formal diagnosis of PMDD from the **first time** you saw a healthcare professional for these symptoms?

- At first contact
- Less than 1 month
- 1 month to 3 months
- 3 months to 6 months
- 6 months to 1 year
- 1 year or more, please specify how long it took __________________________________________________

Display This Question:

If Have you received a formal diagnosis of premenstrual dysphoric disorder (PMDD) from a healthcare... = Yes

At what age did you receive a diagnosis of PMDD?

________________________________________________________________

Display This Question:

If Have you received a formal diagnosis of premenstrual dysphoric disorder (PMDD) from a healthcare... = Yes

|  |
| --- |

Approximately, how many different healthcare professionals did you see before you received a formal diagnosis of PMDD?

- 1
- 2-3
- 4-5
- More than 5, please specify how many different healthcare professionals you had to see __________________________________________________
