# Supplementary material for: Improving care experiences for premenstrual symptoms and disorders in the United Kingdom (UK): a mixed-methods approach
Source: BMC Health Serv Res. 2025 Jan 14;25:70. doi: 10.1186/s12913-024-12140-3 (PMC11730124; doi:10.1186/s12913-024-12140-3)
Supplement: Supplementary file 2 — Supplementary Material 2. [file 12913_2024_12140_MOESM2_ESM.docx]

*Supplementary Table A1. Intercoder reliability evaluation and reporting guidelines (Coffie et al., 2022)*

| **Aspects of Intercoder Reliability** | **Present** | **Justification (If ‘no’ selected)** |
| --- | --- | --- |
| There was a minimum of two coders. | Yes |  |
| At least one coder was more removed from data collection (to address bias). | N/A | Data collection was via an online survey, so no coders were directly involved in data collection. |
| At least one coder had expertise and previous experience with coding qualitative data. | Yes |  |
| If there were multiple participant groups, a minimum of two researchers (coders) coded transcripts from each participant group. | N/A | There were not multiple participant groups. |
| The coders used the same framework for analysis (e.g., inductive, deductive, abductive). | Yes |  |
| Coders focused on shared meaning of *codes through dialogue and consensus. | Yes |  |
| Another coder with expertise in qualitative methods was consulted to resolve outstanding conflicts. | Yes | A third coder (author SB) was available for consultation regarding any unresolved conflicts, but was not required. |
| Coder consensus resulted in a codebook** that was applied when coding the remaining transcripts. | Yes | The codebook was written by author EF after data familiarisation. This codebook was reviewed by author NMK before coding commenced and any necessary revisions to it were made. |

*Key: ** *The code names do not have to be identical, but the meaning of the codes must be the same*; ***In inductive and abductive analyses, coding can be an iterative process; therefore, new codes may be added to the codebook until code saturation is reached*.

Cofie N, Braund H, Dalgarno N. Eight ways to get a grip on intercoder reliability using qualitative-based measures. Can Med Educ J. 2022 May 3;13(2):73-76.

*Supplementary Table A2. Summary of sociodemographic characteristics.*

|  | **Overall (N=339)** | | |
| --- | --- | --- | --- |
|  | n | % | Mean (SD; range) |
| **Age** |  |  | 34.66 (5.88; 18-50) |
| **Gender** | | | |
| Woman | 332 | 97.94 |  |
| Non-binary | 6 | 1.77 |  |
| Other | 0 | 0.00 |  |
| Prefer not to answer | 1 | 0.29 |  |
| **Ethnicity** | | | |
| White or Caucasian | 311 | 91.74 |  |
| Asian (Indian, Pakistani, Bangladeshi, Chinese, or any other Asian background) | 8 | 2.36 |  |
| Black, Caribbean or African | 2 | 0.59 |  |
| Hispanic or Latinx | 4 | 1.18 |  |
| Mixed or multiple ethnic groups | 11 | 3.24 |  |
| Other ethnic group | 2 | 0.59 |  |
| Prefer not to answer | 1 | 0.29 |  |
| **Highest educational attainment** | | | |
| Primary education or below (up to 11 years) | 1 | 0.29 |  |
| Lower secondary education (up to 16 years) | 10 | 2.95 |  |
| Upper secondary education (up to 18 years) | 42 | 12.39 |  |
| Undergraduate degree | 134 | 39.53 |  |
| Postgraduate degree | 142 | 41.89 |  |
| Other | 9 | 2.65 |  |
| Prefer not to answer | 1 | 0.29 |  |
| **Employment status*** | | | |
| Employed (full-time, part-time, self-employed) | 284 | 83.78 |  |
| Unemployed | 16 | 4.72 |  |
| Student | 33 | 9.73 |  |
| Homemaker | 23 | 6.78 |  |
| Maternity/paternity leave or taking time off of work to care for a family member | 8 | 2.36 |  |
| Voluntary work | 5 | 1.47 |  |
| Prefer not to answer | 2 | 0.59 |  |

*Key. * Percentage total may exceed 100 as participants were able to select multiple answer options.*
